# Increased cardiac work provides a link between systemic hypertension and heart failure

**DOI:** 10.14814/phy2.13104

**Published:** 2017-01-13

**Authors:** Alexander J. Wilson, Vicky Y. Wang, Gregory B. Sands, Alistair A. Young, Martyn P. Nash, Ian J. LeGrice

**Affiliations:** ^1^ Auckland Bioengineering InstituteUniversity of AucklandAucklandNew Zealand; ^2^ Department of PhysiologyUniversity of AucklandAucklandNew Zealand; ^3^ Department of Anatomy and Medical ImagingUniversity of AucklandAucklandNew Zealand; ^4^ Department of Engineering ScienceUniversity of AucklandAucklandNew Zealand

**Keywords:** Cardiomyopathy, heart failure, hypertension, hypertrophy, ventricular dysfunction, ventricular remodeling

## Abstract

The spontaneously hypertensive rat (SHR) is an established model of human hypertensive heart disease transitioning into heart failure. The study of the progression to heart failure in these animals has been limited by the lack of longitudinal data. We used MRI to quantify left ventricular mass, volume, and cardiac work in SHRs at age 3 to 21 month and compared these indices to data from Wistar‐Kyoto (WKY) controls. SHR had lower ejection fraction compared with WKY at all ages, but there was no difference in cardiac output at any age. At 21 month the SHR had significantly elevated stroke work (51 ± 3 mL.mmHg SHR vs. 24 ± 2 mL.mmHg WKY;* n* = 8, 4; *P* < 0.001) and cardiac minute work (14.2 ± 1.2 L.mmHg/min SHR vs. 6.2 ± 0.8 L.mmHg/min WKY;* n* = 8, 4; *P* < 0.001) compared to control, in addition to significantly larger left ventricular mass to body mass ratio (3.61 ± 0.15 mg/g SHR vs. 2.11 ± 0.008 mg/g WKY;* n* = 8, 6; *P* < 0.001). SHRs showed impaired systolic function, but developed hypertrophy to compensate and successfully maintained cardiac output. However, this was associated with an increase in cardiac work at age 21 month, which has previously demonstrated fibrosis and cell death. The interplay between these factors may be the mechanism for progression to failure in this animal model.

## Introduction

Heart failure is a condition in which the heart is unable to pump enough blood to meet the metabolic demands of the body. The worldwide prevalence of heart failure is more than 23 million people (Roger 2013), and despite continued research and improved treatment, it continues to be a significant medical burden. Heart failure patients have a 3‐year mortality rate of over 20% for patients with heart failure with preserved ejection fraction (HFpEF), and over 30% for those with heart failure with reduced ejection fraction (HFrEF) (MAGGIC 2012). Half of heart failure patients require readmission to hospital within 6 month (Desai and Stevenson 2012), significantly contributing to costs in the healthcare sector (Braunschweig et al. 2011). Heart failure is the result of cardiac remodeling, which is the progressive rearrangement of both the myocardial tissue structure and ventricular geometry. Cardiac remodeling is a complex process and there is ongoing debate as to whether HFpEF and HFrEF are disparate entities or part of a single spectrum of disease (Borlaug et al. 2011; De Keulenaer et al. 2011). In order to improve heart failure treatments, a better understanding of the pathophysiology of cardiac remodeling is required.

The spontaneously hypertensive rat (SHR) is used as an animal model of heart failure because its progression of cardiac remodeling toward heart failure is reportedly similar to that seen in humans (Trippodo and Frohlich 1981; Doggrell and Brown 1998). Previous findings show that SHRs initially have normal left ventricular (LV) geometry, but develop concentric hypertrophy and eventually progress toward eccentric hypertrophy and systolic dysfunction (LeGrice et al. 2012). Although alterations of geometry and function in the SHR have been described previously, these descriptions of age‐tracked changes have been obtained using echocardiography (Bing et al. 1995; Slama et al. 2004; LeGrice et al. 2012), which has known limitations when used to quantify LV mass and volume (Bottini et al. 1995). MRI is the gold‐standard technique for cardiac imaging and enables precise three‐dimensional (3D) measurement of ventricular volume and function throughout the cardiac cycle. MRI has been used previously to study cardiac function in the SHR (Wise et al. 1998, 1999; Dodd et al. 2012), however, these studies were limited to early ages (2–3 month) and thus did not characterize the development of significant cardiac remodeling.

The purpose of this study was to use MRI to quantify the changes in ventricular geometry and mechanical function that occur through the lifetime of the SHR, and to compare to those of the Wistar‐Kyoto (WKY) rat as control. In addition to the finding that aged SHRs show a reduced myocyte to capillary ratio (LeGrice et al. 2012), it has also been shown that cardiac efficiency, the ratio between work generated per unit myocardial mass and oxygen consumed, correlates inversely with heart mass (Han et al. 2015). We propose that the SHR initially compensates for increased stroke work resulting from increased mean arterial pressure by increasing cardiac mass, but that this hypertrophic response is maladaptive. We also propose that measures of ejection fraction derived from MRI can capture differences between the SHR and WKY groups that are not reflected in echocardiographic estimates of fractional shortening.

Although the SHR is a well‐studied animal model, there has been no comprehensive assessment of cardiac remodeling and ventricular function through the lifetime of the SHR using MRI. The lack of accurate measurements of ejection fraction and stroke volume contributes to the lack of clarity regarding where the SHR fits on the spectrum of heart failure. In this study, cardiac geometry was assessed by estimating LV mass, volume and indices of hypertrophy using 3D model‐based analysis of cardiac MRI. Model‐based analysis involves customizing a finite element model of the LV to MR images, which allows fast and accurate calculation of LV geometry and function (LeGrice et al. 2012). Ventricular function was assessed through measurement of mean arterial pressure and MRI‐derived ejection fraction, cardiac output and cardiac minute work. In this study, in vivo estimates of cardiac work were made possible by combining MRI‐derived estimates of LV volume with measurements of mean arterial pressure.

## Methods

### Animals and procedures

This study was approved by the Animal Ethics Committee of the University of Auckland and conforms to the National Institutes of Health Guide for the Care and Use of Laboratory Animals (NIH Publication No. 85‐23).

The SHR and its corresponding control animal type, WKY, (Harlan Laboratories, Indianapolis, IN) were studied at three ages: 3 month, 14 month, and 21 month. All rats were weighed weekly and had unrestricted access to food and water. All rats were male. From the initial sample of 38 SHRs and 16 WKYs, 13 SHRs, and 2 WKYs died before they could be studied. Experiments were either “terminal” or “recovery”. For terminal studies, animals underwent MR imaging, and were then killed using cervical dislocation. Recovery animals underwent MR imaging under anesthetic and were subsequently revived. These animals underwent terminal studies at a later time‐point. The number of animals studied at each time‐point is included in the caption of Table 1.

**Table 1:**
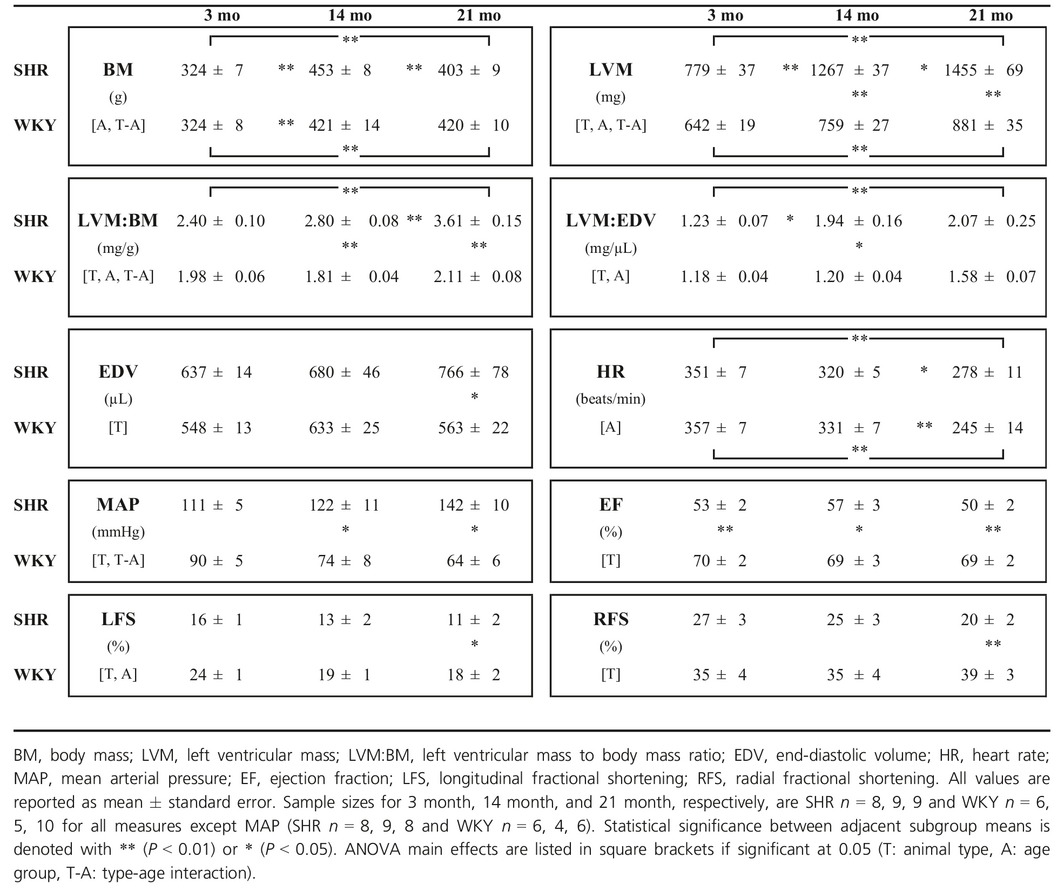
Hemodynamic and morphological measurements

### In vivo pressure measurement

Animals were anesthetized using a face mask with 2–4% isoflurane in air, and the delivery was adjusted in response to respiratory rate. Recovery animals underwent tail‐cuff plethysmography while under anesthetic to determine mean arterial pressure (Buñag and Butterfield 1982) prior to MRI. In animals undergoing terminal studies, the right internal carotid artery was exposed and a pressure transducer (Model SPR‐320, Millar Instruments, Houston, TX) was inserted and advanced to the aortic arch prior to MRI. Mean arterial pressure was derived from pressure traces recorded at this location. Tail‐cuff plethysmography measurements were taken during recovery studies for all 3 month WKY (*n *=* *6), all 3 month SHR (*n *=* *8), all 14 month WKY (*n *=* *4), and some of the 14 month SHR (*n *=* *4). Arterial pressure transducer measurements were obtained during terminal studies for some 14 month SHR (*n *=* *5), all of the 21 month SHR (*n *=* *8), and all of the 21 month WKY (*n *=* *6). At each time‐point, we obtained measurements from a sample of animals from each animal group. We re‐sampled 6 of 14 WKYs, but only 1 of 25 SHRs due to poor survival rate. In the SHR group, 13 of 34 animals died before they could be studied, which may have biased our SHR group results at 21 month toward healthier animals. In the WKY group, 2 of 18 animals died without being studied.

### Magnetic resonance imaging

MR imaging was performed using a Varian 4.7T magnet with a UnityInova spectrometer. A birdcage volume coil (72 mm inner diameter) was used for both transmitting and receiving. An animal monitoring system (Small Animal Instruments Inc., Stony Brook, NY) enabled image acquisition to be gated to ECG and respiration. Throughout imaging, the core body temperature of the animal was maintained at 35–38°C by directing a regulated stream of warm air over the animal, and both heart rate and body temperature were recorded.

Cine MR images at 3 long‐axis (angular spacing = 60°) and 6 short‐axis slices (spacing = 0.6 mm–1.0 mm) were acquired to ensure full coverage of the LV. Each slice was imaged at 18 evenly spaced time‐points through the cardiac cycle. T1‐weighted gradient‐echo cine acquisitions used the following parameters: repetition time, TR = 2 ×  R‐R interval, ~ 280 ms ‐ 360 msec; echo time, TE = 2.2 msec; cardiac phases = 20; flip angle = 20°; slice thickness = 2 mm; averages = 2, field of view = 60 mm × 60 mm; matrix = 128 pixels × 128 pixels; gap between slices = 0.6 mm–1.0 mm according to the size of the heart.

### MR image analysis

The images were analyzed using the Cardiac Image Modelling software (CIM version 6.1, University of Auckland) (Young et al. 2011). A 3D finite element model of the LV was interactively customized to each image frame throughout the cardiac cycle for each animal. Prior to model customization, key landmark points (the centroid of the LV base, the centroid of the LV apex, insertion points of the right ventricular free wall with the interventricular septum, and the position of the mitral valve plane) were identified to register an initial model to the images. For each image frame, the endocardial and epicardial surfaces of the initial LV model were customized to all slices using interactive guide‐point modeling. Fourier‐series filtering in the time domain was also incorporated to ensure a smooth transition of the time‐varying 3D models throughout the whole cardiac cycle. The resulting 3D models provided LV geometric and functional measures such as LV mass, ejection fraction, end diastolic volume (EDV), end systolic volume (ESV), and stroke volume. Longitudinal and radial (both anterior‐posterior and septal‐lateral) fractional shortening were also calculated from the 3D models using customized software. In addition to the aforementioned indices, stroke work was calculated as the product of stroke volume and mean arterial pressure. Cardiac minute work, defined as the energy expended per minute, was calculated as the product of stroke work and heart rate.

### Statistical analysis

Data were analyzed using the statistical package R (Version 3.0.2, R Foundation for Statistical Computing, Vienna, Austria). For each measure, an analysis of variance (ANOVA) was performed using a two‐way mixed factor model including age (A) as a measure with three levels (3 month, 14 month, 21 month), and animal type (T) with two levels (SHR and WKY), as well as the interaction term (T‐A). Post hoc comparisons were performed using the Tukey Honest Significant Difference procedure. These comparisons between age/type subgroups are presented in Table 1 where significant. A significance level of *P* < 0.05 was used for statistical comparisons within figures. All values are reported as mean ± standard error.

## Results

With reference to Table 1, SHR developed both hypertension and hypertrophy, which is consistent with previous findings (Cingolani et al. 2003; LeGrice et al. 2012; Han et al. 2015). Body mass was largest at 14 month for both animal types and no significant differences between animal types were observed. SHR group LV mass was not significantly different from the WKY group at 3 month, but significantly greater than control at 14 and 21 month. These differences were present both without and with correction for body mass. Hypertrophy in the SHR at 21 month was evidenced by an increase in LVM/tibia length compared with WKY (SHR 27.4 ± 1.0 mg·mm^−1^, WKY 16.5 ± 0.5 mg·mm^−1^, *P* < 0.001). SHRs exhibited LV dilatation at 21 month as demonstrated by the significantly greater EDV compared with control. The ratio of LV mass to EDV has previously been used as an index of concentric hypertrophy (Fonseca et al. 2003; Oxenham et al. 2003; Lumens et al. 2006). At 14 month, the LV mass to EDV ratio of SHRs was significantly larger than WKY, consistent with concentric hypertrophy, but at 21 month there was no significant difference between animal types.

Based on the MRI‐derived EFs, the SHRs had EFs that were significantly lower than controls at all time‐points, including the 3 month time‐point when SHRs did not have significant hypertension compared to control. Conversely, longitudinal fractional shortening and radial fractional shortening measures showed significant differences between animal types only at 21 month.

**Figure 1:**
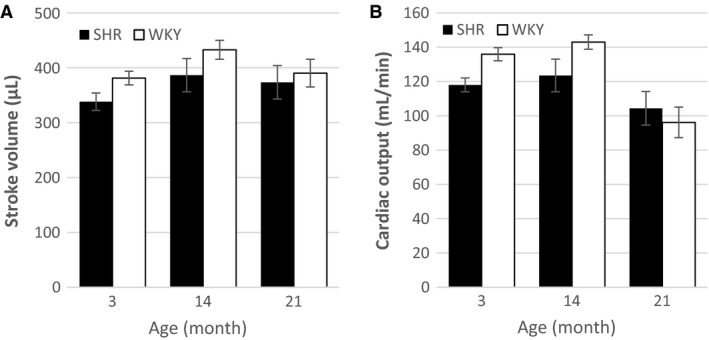
Measures of ventricular pumping. **A** Stroke volume and **B** cardiac output for SHR and WKY at 3 month, 14 month, and 21 month (SHR *n* = 8, 9, 8 and WKY *n* = 6, 4, 6). Data are visualized as mean ± standard error for each animal‐age subgroup. No significant differences between animal types were observed.

**Figure 2:**
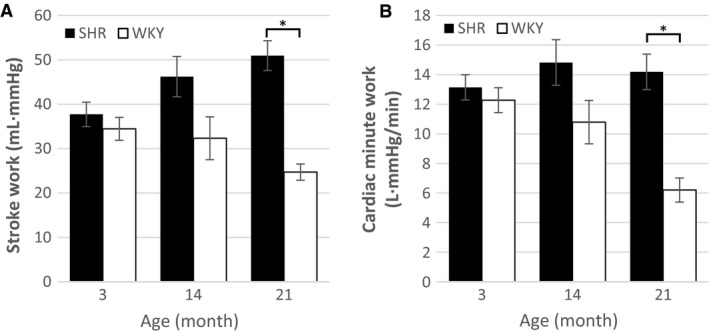
Measures of ventricular work. **A** Stroke work and **B** cardiac minute work for SHR and WKY at 3 month, 14 month, and 21 month (SHR *n* = 8, 9, 8 and WKY *n* = 6, 4, 6). Data are visualized as mean ± standard error for each animal‐age subgroup. At 21 month, SHR had significantly greater stroke work and cardiac minute work than WKYs.

Although EF was impaired in the SHRs, no significant differences between animal types were observed for either stroke volume or cardiac output (Fig. 1). At 3 month and 14 month, the SHRs performed similar amounts of stroke work and cardiac minute work compared to control (Fig. 2). However, at 21 month, both measures of work were significantly larger in the SHR group, which reflects the increased mechanical energy required to maintain cardiac output in the presence of pressure overload.

## Discussion

This study characterized global cardiac structural and functional remodeling throughout the lifetime of the SHR. MRI was used to evaluate cardiac structure and function, since it is a robust imaging technique and provides the best available in vivo measurements for 3D cardiac geometry and function. Previous MRI studies of the SHR have been performed in animals up to 7 month of age (Laurent et al. 1995; Wise et al. 1998, 1999; Dodd et al. 2012), but these studies do not include ages that have been previously identified to coincide with key cardiac remodeling states, such as compensated hypertrophy at 12 month and progression to decompensated failure at 18–24 month. Comparable lifetime studies have been limited to echocardiography‐derived measurements (Bing et al. 1995; Slama et al. 2004; LeGrice et al. 2012), which are inherently less reliable than MRI (Bottini et al. 1995). From accurate measurements of stroke volume, cardiac output, and mean arterial pressure, we were able to estimate measures of myocardial work and gain mechanistic insights into ventricular remodeling.

This longitudinal MRI study adds to the established literature investigating the SHR animal model. One echocardiographic study showed no difference in FS between SHR and WKY at any age (Slama et al. 2004), whereas another found differences in FS past 18 month (LeGrice et al. 2012). Our MRI results show differences in EF from 3 month, which is consistent with a previous study (Wise et al. 1998). Other studies (Shimamoto et al. 1982; LeGrice et al. 2012) have shown differences in blood pressure from 3 month, and although our anaesthetized MAP values showed no difference between groups, our measurements of systolic blood pressure in awake animals showed that SHRs were hypertensive compared with WKY at 4 month (SHR 201 ± 3 mmHg, WKY 151 ± 4 mmHg, *P *<* *0.01). Also, hypertrophy has been observed in the SHR at 3 month (Shimamoto et al. 1982; Wise et al. 1998), however, based on our LVM/BW measurements, we only had weak evidence that our SHRs were hypertrophic at 3 month (*P* < 0.1).

Diastolic dysfunction is thought to be an important characteristic of heart failure progression, particularly related to hypertensive cardiomyopathy (Lamb et al. 1999). Previous studies have shown that, compared to age‐matched controls, 10 month SHRs exhibited diastolic dysfunction (Cingolani et al. 2003), and 12 month isolated SHR hearts were stiffer (LeGrice et al. 2012). Concentric hypertrophy and diastolic dysfunction are traditionally associated with a small EDV, while a large EDV is associated with a dilated ventricle and systolic dysfunction. Measurements of mean arterial pressure confirmed that the SHRs were hypertensive from 14 month onwards, and this was accompanied by hypertrophy as indicated by the elevated ratio of LV mass to body weight. Our analysis revealed significant differences in the LV mass to EDV ratio that are consistent with the development of concentric type hypertrophy in the SHR, which is often associated with diastolic dysfunction. In humans it has been reported that patients with HFpEF have a significantly larger LV mass to EDV ratio than patients with HFrEF (Santhanakrishnan et al. 2012). LV mass to EDV ratio was maintained in the SHR at the 21 month time‐point, indicating concentric hypertrophy. Also, at this time‐point we observed ventricular dilatation in the SHR, in addition to reduced EF. The combination of ongoing low EF and progressive hypertrophy in the SHR makes it difficult to place on the spectrum of human heart failure.

Since our MRI protocol provided images encompassing the entire LV, our study enabled accurate measurement of cardiac ejection fraction and stroke volume. Mean ejection fraction was found to be lower in the SHR compared with WKY at all time‐points. Previous studies of the SHR used echocardiography, which provides estimates of fractional shortening (a 1D approximation of ejection fraction) that can be converted to estimates of ejection fraction by assuming an idealized ventricular geometry. To provide a comparison with echocardiography, we calculated the mean radial fractional shortening and longitudinal fractional shortening. These measures were observed to be significantly lower in the SHR at only the 21 month time point. In contrast, the ejection fraction derived from MRI revealed significant differences in systolic function at all ages, indicating that the 1D fractional shortening indices did not sufficiently reflect the 3D differences.

Although ejection fraction was lower in the SHR compared to WKY, stroke volume was similar due to the larger EDV. Since there were no observed differences in heart rate between animal types, these similarities in stroke volume corresponded with similar estimates of cardiac output. The larger EDV observed in SHR compared to control is consistent with a compensatory effect that maintains cardiac output in the face of reduced ejection fraction. This compensatory mechanism may increase myocardial fibrosis in aging SHRs leading to increased chamber stiffness (LeGrice et al. 2012) and increased end‐diastolic pressure, which promotes eccentric hypertrophy and chamber enlargement (Grossman et al. 1975). Even though the age‐matched groups of SHRs and WKYs had similar stroke volumes and cardiac outputs, the 21 month SHRs had higher MAP and therefore performed more cardiac minute work to achieve normal cardiac output. It has been previously shown that SHRs have a lower ratio of capillaries to myocytes (LeGrice et al. 2012). This will likely lead to inefficient oxygen delivery, which, when combined with the increased energetic demand, may result in ischemic myocytes and cell death. Myocyte death would give rise to fibrosis, which would exacerbate the downward spiral into heart failure.

We did not observe low cardiac output in the SHR at 21 month, an age at which we expected to observe signs of failure in the SHR. As 13 of 34 SHRs died before they could be studied, it is likely that our SHR group data at 21 month are biased toward healthier animals and do not capture the failure state of the SHR animal model. The reduced efficiency (Han et al. 2015) coupled with the myocardial fibrosis (LeGrice et al. 2012) observed in the aged SHR, are likely to be important factors that would lead to compromised ventricular function. Metabolic effects have previously been shown to precede mechanical dysfunction in the SHR (Hernandez et al. 2013), suggesting that SHR ventricular function may decline soon after maximal cardiac work at 21 months of age. In HFpEF patients, an increase in stroke volume is accompanied by a larger increase in stroke work than is seen in control (Kawaguchi et al. 2003), which is consistent with our stroke work measurements in aged SHRs.

Although we believe that these results are valuable contributions to the SHR literature, there are a number of limitations worth noting. Although MRI gives good estimates of EF as its 3D coverage allows accurate calculation of cavity volumes, echocardiography can give better measurements of diastolic function, such as isovolumetric relaxation time and deceleration time, due to its higher temporal resolution. Another limitation of this study was that in order to obtain cardiac MRI the animals were fully anesthetized. The anesthetic level may have affected SHRs and WKYs differently, which should be taken into account when interpreting these results. In this study, only male rats were used. It has been shown previously that gender plays a role in development of hypertension in the SHR (Reckelhoff et al. 2000) and this difference may, in general, affect the applicability of our results to human HF.

As the SHR is a chronic animal model, the measurements of progressive cardiac remodeling were confounded by other health‐related effects of aging. However, this problem is also present in investigating typical human HF. Another limitation of the SHR is that they are hypertensive by young adulthood rather than middle age as is typically observed in humans. Concentric hypertrophy observed in the SHR could be considered as compensatory remodeling in response to chronic hypertension, as is believed to be the case in the human condition (Gaasch and Zile 2004). However, the utility of the SHR for investigating human hypertensive heart disease has been brought into question by the demonstration that a genetic locus in the SHR influences LV mass independent of blood pressure (Innes et al. 1998). In contrast, observations of end‐stage structural changes in SHR and human hypertensive heart disease show comparable morphological changes (Rossi 1998; LeGrice et al. 2012), thus the issue remains unresolved. One possible way forward is to examine cardiac remodeling and ventricular function in the presence of altered pressure overload, for instance by treating the SHR with an angiotensin‐converting enzyme inhibitor. If in fact SHR hypertrophy is independent of hypertension, SHR treated with an angiotensin‐converting enzyme inhibitor may develop energetically inefficient hypertrophy in the absence of pressure overload.

In summary, we have used cardiac MRI to make longitudinal measurements of geometric and functional remodeling in the SHR heart, and compared these to age‐matched control hearts. SHRs were found to have impaired systolic function, but were able to successfully compensate and maintain cardiac output. However, this was associated with an increase in both cardiac work and LV mass, which may be indicative of adverse feedback in cardiac structural and functional remodeling that ultimately leads to heart failure. We also found that 1D fractional shortening measurements may not sufficiently capture differences in contractile function between animal types that MRI‐based estimates of ejection fraction can capture. These findings provide important insight into the progression from compensated hypertrophy to decompensated heart failure.

## Conflict(s) of Interest

None.
